# Gene Therapy Based on Mesenchymal Stem Cells Derived from Adipose Tissue for the Treatment of Obesity and Its Metabolic Complications

**DOI:** 10.3390/ijms24087468

**Published:** 2023-04-18

**Authors:** Marta Lopez-Yus, Maria Pilar García-Sobreviela, Raquel del Moral-Bergos, Jose M. Arbones-Mainar

**Affiliations:** 1 Adipocyte and Fat Biology Laboratory (AdipoFat), Translational Research Unit, University Hospital Miguel Servet, 50009 Zaragoza, Spain; 2 Instituto Aragones de Ciencias de la Salud (IACS), 50009 Zaragoza, Spain; 3 Instituto de Investigación Sanitaria (IIS) Aragon, 50009 Zaragoza, Spain; 4 CIBER Fisiopatología Obesidad y Nutrición (CIBERObn), Instituto Salud Carlos III, 28029 Madrid, Spain

**Keywords:** adipose tissue-derived mesenchymal stem cell, ADMSC, cell therapy, gene editing, obesity

## Abstract

Obesity is a highly prevalent condition often associated with dysfunctional adipose tissue. Stem cell-based therapies have become a promising tool for therapeutic intervention in the context of regenerative medicine. Among all stem cells, adipose-derived mesenchymal stem cells (ADMSCs) are the most easily obtained, have immunomodulatory properties, show great ex vivo expansion capacity and differentiation to other cell types, and release a wide variety of angiogenic factors and bioactive molecules, such as growth factors and adipokines. However, despite the positive results obtained in some pre-clinical studies, the actual clinical efficacy of ADMSCs still remains controversial. Transplanted ADMSCs present a meager rate of survival and proliferation, possibly because of the damaged microenvironment of the affected tissues. Therefore, there is a need for novel approaches to generate more functional ADMSCs with enhanced therapeutic potential. In this context, genetic manipulation has emerged as a promising strategy. In the current review, we aim to summarize several adipose-focused treatments of obesity, including cell therapy and gene therapy. Particular emphasis will be given to the continuum from obesity to metabolic syndrome, diabetes, and underlying non-alcoholic fatty liver disease (NAFLD). Furthermore, we will provide insights into the potential shared adipocentric mechanisms involved in these pathophysiological processes and their remediation using ADMSCs.

## 1. Introduction

According to the definition by the Obesity Medicine Association, “Obesity is a chronic, relapsing, multifactorial, neurobehavioral disease, wherein an increase in body fat promotes adipose tissue (AT) dysfunction and abnormal fat mass physical forces, resulting in adverse metabolic, biomechanical, and psychosocial health consequences” [1]. This definition encompasses three main consequences of obesity, including metabolic disturbances typically associated with high blood sugar levels and altered blood lipids. These alterations lead to an increased risk of cardiovascular disease, cancer, and dysfunction of multiple organs, such as the pancreas and liver [2].

In the last 40 years, the prevalence of obesity has skyrocketed globally. In 2015, more than 600 million adults were obese; a high body mass index (BMI) accounted for 4.0 million deaths worldwide [3]. However, the public health war against obesity has failed to reduce the prevalence of obesity, often producing unintended consequences, such as an excessive weight concern among the population, which can lead to a negative body image and eating disorders [4]. Consequently, various alternative strategies have emerged to minimize the health-related consequences of obesity.

Currently, bariatric surgery remains the most effective and cost-saving treatment for obesity and its complications [5]; although, because of its complexity, it seems unable to reduce the obesity pandemic expansion. On the other hand, some pharmacological strategies targeting the energy balance regulatory system are now reaching clinically relevant reductions in body weight [6,7]. The appearance of new tools and techniques of genetic engineering as well as advances in understanding the molecular basis of obesity and its metabolic complications have given rise to new precision medicine approaches targeting adipose tissue as an anti-obesity therapy. These innovative approaches include cell-based therapy and gene therapy for obesity.

This narrative review will focus on noninvasive approaches for the adipose-focused treatment of obesity, including cell therapy and gene therapy. Particular emphasis will be given to the continuum from obesity to metabolic syndrome, diabetes, and underlying non-alcoholic fatty liver disease (NAFLD). We aim to provide insight into the potential shared adipocentric mechanisms implied in these pathophysiological processes and their remediation using adipose-derived mesenchymal stem cells (ADMSCs).

## 2. Adipose Tissue-Derived Mesenchymal Stem Cells

### 2.1. Adipose Tissue and the Expandability Hypothesis

It is currently accepted that white adipose tissue (WAT) is not a mere energy reservoir and is considered an endocrine organ producing various cytokines (adipokines) and other metabolites to control systemic energy expenditure [8]. Adipose tissue is extraordinarily heterogeneous in terms of its composition and body distribution. Subcutaneous white adipose tissue (scWAT) is located under the skin and is the largest storage site for excess lipids. We know there is an individual limit on the capacity to store lipids in scWAT (i.e., the adipose tissue expandability hypothesis [9]). The expansion of scWAT is determined by two main processes: the differentiation of new mature adipocytes from their precursors, known as mesenchymal stem cells (MSCs), and the ability to enlarge from those already formed adipocytes [10]. This adipogenic process is coordinated by the differential expression of genes, proteins, microRNAs, and metabolites from different cell types.

When scWAT reaches its maximal storage capacity, adipose tissue fails to store lipids appropriately, redirecting this lipid flux to other organs where it is accumulated as ectopic fat and causes lipotoxicity and inflammation [11]. This ectopic accumulation occurs primarily in the visceral adipose tissue (vWAT) and liver, leading to visceral obesity and liver steatosis [12].

### 2.2. Mesenchymal Stem Cells

MSCs are multipotent stroma cells characterized by the capacity of self-renewal and the ability to differentiate into cell types of mesodermal origin, including adipocytes, osteoblast, and chondrocytes. These cells can be obtained from multiple sources, including adult bone marrow, adipose tissue, peripheral blood, and various neonatal birth-associated tissues [13,14].

MSCs have emerged as a promising therapeutic strategy for various diseases in recent years. Their clinical relevance was initially based on their tissue regeneration capacity, but discovering their paracrine properties has largely extended the range of therapeutic applications [15]. Several characteristics favor their use in treating a wide range of conditions [16]. For instance, MSCs can migrate to a wide range of tissues, specialty inflammatory and pathological sites, because injured tissues secrete chemokines that attract MSCs [17]. Additionally, MSCs possess immunomodulatory properties which are mediated by cell-cell interactions and the secretion of soluble paracrine factors [18]. MSCs can modulate immune system cells’ proliferation and activation, inhibiting CD4+ and CD8+ T cells’ proliferation, regulatory T (Treg) cells’ activation, inducing M2 macrophage polarization, suppressing the function of dendritic cells (DCs), ameliorating B-cells and natural killers’ (NK) proliferation, and decreasing macrophages’ and neutrophils’ infiltration into inflammation sites [19,20].

Other characteristics that make MSCs good candidates for cell therapy are their potential to differentiate into multiple lineages and their ability to be easily expanded ex vivo while retaining their original lineage differentiation commitment [16]. After transplantation, MSCs can differentiate into chondrocytes and undergo chondrogenesis [21]; cardiomyocyte-like cells that integrate into host tissue [22]; hepatocyte-like cells (HLCs) that contribute to liver regeneration [23]; or into insulin-producing cells (IPCs) that secrete insulin [24].

Therefore, these cells are interesting therapeutic tools to target damaged tissues and act as a reservoir of growth factors and immuno-modulatory molecules.

### 2.3. Adipose Tissue as Source of MSCs for Cell Therapy

MSCs obtained from adipose tissue have been proposed as an exciting tool for cell therapy due to their easy isolation and abundance. The characteristics of ADMSCs greatly vary depending on the type and anatomical region of AT. Thus, scWAT is the primary source of ADMSCs, mainly because more than 2% of this tissue is constituted by the stroma vascular fraction (SVF) [25]. Compared with bone marrow (BM), where the MSCs fraction only constitutes 0.001% to 0.1%, AT can provide up to 500-fold more MSCs than an equivalent quantity of BM aspirate, reinforcing that AT is the most abundant and efficient source of MSCs [26].

Although most of the characteristics of ADMSCs are similar to MSCs obtained from other sources, some differences also exist. For instance, several investigations have shown that ADMSCs are stronger immunomodulators that can adapt better to oxidative stress or hypoxia-induced apoptosis and have a greater angiogenetic capacity when exposed to unfavorable conditions [27]. These advantages, compared with other MSCs, are mainly explained by the release of higher levels of pro-inflammatory and anti-inflammatory cytokines, including interferon γ (IFN-γ), interleukins (IL-6, IL-8), and transforming growth factor (TGF-β). Moreover, ADMSCs secrete a higher quantity of growth factors such as granulocyte macrophage colony-stimulating factor (GM-CSF), granulocyte colony-stimulating factor (G-CSF), nerve growth factor (NGF), or insulin-like growth factor 1 (IGF-1) that makes them proliferate better than other MSCs [24,28,29]. Additionally, ADMSCs are more suitable for regenerative medicine, as they differentiate better into β-cells, muscle cells, or cardiomyocytes [30,31,32].

Overall, their abundance, easy isolation, and superior characteristics have boosted interest in using ADMSCs for clinical applications.

## 3. ADMSCs in the Treatment of Obesity and Its Metabolic Complications

**Figure 1:**
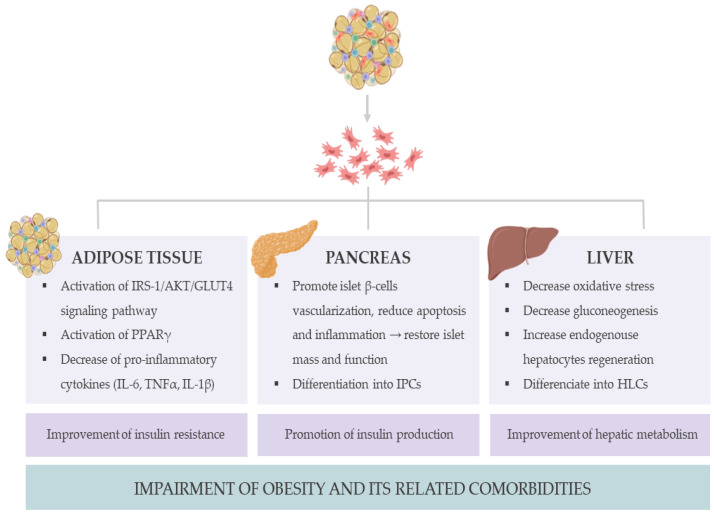
The mechanisms of action of ADMSC on obesity treatment. The transplantation of ADMSC reduces adipose tissue inflammation, restores glucose homeostasis by improving insulin sensitivity and promoting insulin production, and reverses liver steatosis. Abbreviation: ADMSCs, adipose-derived mesenchymal stem cells; AKT, serine/threonine kinase 1; GLUT4, glucose transporter 4; HLCs, hepatocyte-like cells; IL-1β, interleukin 1β; IL-6, interleukin 6; IPCs, insulin-producing cells; IRS-1, insulin receptor substrate 1; PPAR-γ, peroxisome proliferator-activated receptor gamma; and TNF-α, tumor necrosis factorα.

Various interventions have been proposed for obesity and its related disorders in clinical settings. However, effective therapies to prevent and remedy obesity and its comorbidities are still lacking [33]. In this context, ADMSC therapy has emerged as a promising strategy. The results obtained in animal models have confirmed their effects on weight loss, changes in adipose tissue composition, and improvement of related comorbidities such as diabetes or NAFLD. The most promising in vivo results are reviewed below and summarized in Table 1 and Figure 1.

### 3.1. Body Composition and Weight Loss

Weight loss is a critical step in treating obesity and its related complications. Numerous in vivo experiments have shown the efficiency of ADMSCs in weight loss and the reduction in body fat mass. Cao et al. investigated the effects of ADMSCs on body weight and composition in a mouse model of high-fat diet (HFD)-induced obesity [34]. A single intravenous infusion of ex vivo expanded syngeneic ADMSC significantly reduced body weight and triglyceride levels. In line with these findings, Tung-Qian Ji et al. demonstrated that two episodes of systemic human ADMSC transplantations effectively decrease body weight in mice [35]. Likewise, Lee et al. showed that the transplantation of human ADMSC, MSC-derived brown adipocytes (M-BA), and MSC lysate into obese mice reduced body weight and hyperlipidemia [36]. In a similar fashion, Liu et al. used leptin receptor-deficient (db/db) mice and diet-induced obesity (DIO) mice to compare the effects of the administration of MSCs obtained from different sources: AT and an umbilical cord (UC) [37]. Their results showed that three weeks of ADMSC administration blocked body weight gain and AT weight was decreased in both obesity models. In contrast, UC-MSC administration had little or no effect on the animals’ body weight and AT weight, suggesting that MSCs isolated from different sources may differ in their physiological functions.

However, another study by Shang et al. showed that treating obese mice with ADMSC did not change body weight, although it reduced adipocyte hypertrophy [38]. Similarly, Jaber et al. showed a significant reduction in body fat mass, despite no change in body weight [39]. Interesting results were also obtained by Shree et al. [40]: HFD-fed mice were treated with human ADMSCs or ADMSCs preconditioned with metformin. It turned out that animals administrated with ADMSCs alone did not change body weight, but significant weight loss was reported in the metformin-preconditioned ADMSC mice.

Distinct animal models, different adipose tissue depots selected for ADMSC isolation, and differing administration methods could explain the discrepancies between the studies. However, all the in vivo experiments seem to suggest that ADMSC therapy could effectively alter body composition [34].

### 3.2. Diabetes

Obesity is associated with impaired glucose tolerance, often leading to type 2 diabetes mellitus (T2DM). T2DM, which constitutes 90% to 95% of all cases of diabetes, is characterized by decreased insulin sensitivity in peripheral tissues such as adipose tissue, skeletal muscles, or liver, as well as a progressive loss of proper insulin secretion [41]. T2DM reduces functional capacities and quality of life, dramatically increasing morbidity and premature mortality [42]. Therefore, novel therapeutic strategies can help to treat diabetes and reverse long-term complications effectively.

In this context, ADMSC-based therapy has emerged as a promising strategy. Several studies have proved its capacity to maintain glucose homeostasis and reduce insulin resistance in obesity models. For instance, Lee et al. showed that multiple administrations of ADMSCs upregulated the expression of GLUT4 in AT and skeletal muscle and reduced gluconeogenesis in the liver of HFD-fed mice, reducing insulin resistance [36]. Moreover, ADMSC-based treatments altered the adiponectin-to-leptin ratio and regulated the expression of *Ppara* and *Pparg*, which maintain energy homeostasis in major metabolic tissues. In line with these findings, other studies in similar models demonstrated that DIO mice receiving ADMSC injections improved glucose tolerance and insulin sensitivity, as indicated by a significant decrease in glucose levels [34,38,39,43]. Ishida et al. developed and transplanted ADMSC sheets subcutaneously in diabetic mice fed an HFD and high sucrose diet (HSD). ADMSC transplantation significantly increased adiponectin and decreased tumor necrosis factor- α (TNF-α) plasmatic levels, ultimately improving mice glucose tolerance [44].

Additionally, ADMSC has been reported to differentiate into insulin-producing cells (IPC). Timper K et al. reported for the first time the potential of ADMSCs to derive IPC, but initially, these cells did not secrete insulin [45]. Later, ex vivo experiments performed by Dave et al. showed that ADMSCs collected from scWAT and cultured in a specific differentiation medium began to express *Pdx1, Pax6*, and *Isl1*, all essential factors for the reprogramming of nonpancreatic cells to fully functional β-cells. They also reported that the stimulation of these cells led to the secretion of insulin [46]. In line with these findings, Karaoz et al. demonstrated that the ability of ADMSCs to differentiate into IPC was higher than MSCs isolated from BM, indicating AT as a better cell source for cell-based therapy to restore the metabolic complications of diabetes [24].

The potential of ADMSCs to produce insulin was also tested in vivo by Nam et al. [47]. They showed that the transplantation of IPC differentiated from ADMSCs underneath the kidney capsule of T2DM mice significantly increased circulating insulin and reduced hyperglycemia, as well as ameliorated triglycerides (TG), free fatty acids (FFA), and IL-6 serum levels. Similar outcomes were reported by Liang et al. who developed a chemical-based protocol that increased the efficiency of IPC generation, which, when transplanted, significantly mitigated hyperglycemia in diabetic rats [48]. In this context, Chandra et al. explored the potential of ADMSCs to generate pancreatic hormone-expressing islet-like cell aggregates (ICAs) from murine epididymal MSCs [49]. These cells expressed pancreatic hormones such as insulin, glucagon, or somatostatin and had secretory capacity. The transplantation of calcium alginate-encapsulated ICAs into diabetic mice restored normoglycemia within two weeks, demonstrating the feasibility of using ADMSCs as a source of autologous stem cells to differentiate into the pancreatic lineage.

The dual action of ADMSCs in treating diabetes, regulating insulin sensitivity in peripheral tissues, and restoring β-pancreatic cells’ function was demonstrated by Tung-Quian et al. [35]. They showed that two episodes of systemic MSC administration in DIO mice effectively improved glucose homeostasis by targeting the pancreas and insulin-sensitive tissues via site-specific mechanisms. ADMSCs supported pancreatic islet growth by direct differentiation into IPC and by reducing the cytotoxicity of IL-1 and TNF-α secretion. At the same time, the localization of ADMSCs in peripheral tissues improved glucose tolerance, reducing serum levels of adipokines, restoring glycogen storage in hepatocytes, and increasing the expression of the IL-1 receptor antagonist and GLUT4 in skeletal muscles. Taken together, these results suggest that systemic ADMSCs administration ameliorates HFD-induced obesity and restores metabolic balance through multisystemic regulations with some tissue-specific mechanism.

The efficiency of ADMSC therapy has been tested not only in obesity-induced IR mouse models but also in T2DM rat models. Xie et al. injected ADMSCs into streptozotocin (STZ)-induced diabetic rats and observed a decrease in glucose blood levels, an increase in glucose tolerance, and an enhancement in insulin sensitivity [50]. They examined the regulation of hepatic glucose metabolism to address the mechanism involved in this improvement. They observed that ADMSC administration stimulates the phosphorylation of hepatic AMP-activated protein kinase (AMPK) to restore hepatic glucose metabolism in T2DM. In another study, Wang et al. assessed the potential therapeutic effect of ADMSC isolated from HFD/STZ-induced T2DM and db/db mice [51]. Although less proliferative than cells from healthy controls, the intravenous injection of ADMSC from T2DM or db/db increased pancreatic β cell mass and insulin sensitivity while reducing inflammation up to 5 weeks post-infusion. This study offers evidence that ADMSCs from diabetic donors also have some potential for cell therapy in the treatment of insulin resistance and T2DM.

All the above studies prove the potential of ADMSCs for treating early phase T2DM. However, research on the late stages of diabetes is more scarce. For example, Hu et al. induced T2DM in a rat model that mimics the long-term complications of diabetes and administrated ADMSCs [52]. They observed that ADMSC infusion effectively reduced hyperglycemia, restoring pancreatic islet β-cells function and improving IR via the upregulation of GLUT4 in insulin-sensitive tissues. Similar results were reported by Yu et al. [53]. In this study, multiple intravenous infusions of ADMSCs efficiently restored glucose homeostasis, lowered serum lipid levels, and ameliorated the progression of metabolic complications such as chronic kidney disease (CKD), non-alcoholic steatohepatitis (NASH), lung fibrosis, and cataracts in rats with T2DM. Altogether, the results reported thus far demonstrated that ADMSC therapy has an anti-diabetic effect and alleviates not only the early stages but also the long-term complications of T2DM.

### 3.3. NAFLD

NAFLD is rapidly becoming one of the most common liver diseases, with a prevalence of 25% worldwide [54]. It is an umbrella term for a variety of conditions caused by excessive fat accumulation in the form of triglycerides in hepatocytes. Hepatosteatosis represents the first step of the disease. Steatosis can be benign and remain clinically silent. However, in many cases, complications can ultimately lead to steatohepatitis (NASH), fibrosis, cirrhosis, or even hepatocarcinoma [54]. NAFLD is often linked to the development of severe obesity and metabolic syndrome (MetS); it is estimated that more than 75% of patients with obesity are affected by NAFLD [55,56]. The underlying cause of NAFLD is complex and multifactorial. Some key inducers of this condition include the accumulation of lipids in hepatocytes, pro-inflammatory mediators, mitochondria dysfunction, and genetic and epigenetic factors [57]. Although the pathogenesis is well-studied, there is currently a lack of effective therapeutic options for this condition.

Self-renewal capacity, high multilineage potential, and anti-inflammatory and antioxidant properties make ADMSCs a promising alternative for treating NAFLD [23,58]. Several studies have demonstrated that the inflammatory factors secreted by injured liver tissue attract ADMSC to this organ [59,60]. ADMSCs engraft into the recipients’ livers, enhancing hepatocyte proliferation and regeneration and acting as antioxidants. Pan et al. demonstrated that ADMSC transplantation into the liver of HFD-induced NAFLD rats restored the oxidative balance by reducing the content of malondialdehyde (MDA) and increasing the enzymatic activity of antioxidant defense such as superoxide dismutase (SOD), resulting in a reduction in lipid metabolism and an improvement of liver function, attenuating the disease progression of HFD-induced NAFLD [61]. These findings are in line with other investigations that showed that ADMSC therapy ameliorated hepatic oxidative stress through the upregulation of SOD and NADPH quinone oxidoreductase 1 (NQO1) activity and the downregulation of myeloperoxidase (MPO) after liver injury [62].

Furthermore, it has been shown that ADMSCs improve hepatic inflammation, which is a determinant in the progression of NAFLD. In this sense, Ezquer et al. demonstrated that ADMSC administration in obese mice with MetS reduced the levels of fibrosis markers and pro-inflammatory cytokines, although they still present steatosis [59]. In line with these findings, Yamato et al. showed that ADMSC treatment prevented the progression of NASH by suppressing IL-17-mediated inflammation, which was associated with hepatic stellate cell (HSC) activation [63]. In addition, Lee et al. showed that in severe liver fibrosis caused by persistent HFD-feeding, ADMSC transplantation downregulated the expression of pro-inflammatory cytokines as IL-4 or TNF-α while increasing the expression of anti-inflammatory cytokines [36].

Considering these positive results, the immunomodulatory activity of the extracellular vesicles (EV) released by ADMSCs have also been studied. Watanabe et al. used a melanocortin type-4 receptor knockout (Mc4r-KO), a mouse model of NASH with a rapid accumulation of fibrosis. They showed a similar improvement in fibrosis in the groups treated with MSCs and their EVs, as well as a significant increase in anti-inflammatory macrophages in the liver [64].

Another critical characteristic of ADMSCs that makes them interesting candidates to treat liver injury is their capacity to differentiate into hepatocyte-like cells (HLCs) and adopt the hepatocyte phenotype [65,66]. It has been reported that these ADMSC-derived HLCs are able to secret albumin, synthesize glycogen and urea, and have cytochrome P450 (CYP) enzyme activity [66]. In vivo experiments performed by Banas et al. showed that the transplantation of ADMSC-derived HLC restored liver function in a mouse model of acute liver failure [65]. These results concur with another study where the transplantation of HLCs increased the secretion of IL-10, IL-6, and TGF-β, and preserved liver function in a mouse model of liver injury induced by carbon tetrachloride (CCl4) [67]. Of note, some studies showed that although HLCs could improve liver function, these cells lost several major properties and quickly progressed to cell death when transplanted in vivo [68]. In contrast, ADMSCs were not sensitive to the damaged environment and were preserved in the liver longer. Therefore, the autologous transplantation of ADMSCs in vivo seems more efficient than HLC for liver regeneration.

Taken together, the data reported so far indicate the therapeutic potential of ADMSCs to ameliorate the development of chronic liver disease, extending to NAFLD, liver fibrosis, and cirrhosis in animal models.

**Table 1:** The most relevant pre-clinical studies exploring the therapeutic efficiency of ADMSCs in the treatment of obesity and its metabolic complications.

ADMSC Source	Animal Model	Administration of ADMSC	Effects	Reference
Mouse	HFD mice	2 × 10^6^ cells injected intravenously	Reduced body weight Reduced blood glucose levels and increased glucose tolerance Reduced fat cell deposition in the liver	[34]
Mouse	HFD mice	1 × 10^6^ cells injected intraperitoneally	Reduced adipocyte hypertrophy Improved insulin action and metabolic homeostasis Attenuated WAT inflammation	[38]
Mouse	HFD mice	0.5 × 10^6^ cells injected intravenously	Prevented the onset of NASH, alleviated the inflammatory process	[59]
Mouse	HFD and HSD mice	ADMSC sheet transplantation into subcutaneous sites	Improved glucose intolerance Increased plasma adiponectin and decreased tumor necrosis factor-α levels	[44]
Human	HFD mice	4.2 × 10^7^ cells/kg injected intravenously	Decreased body weight Improved glucose tolerance and blood glucose homeostasis	[35]
Human	HFD mice	1 × 10^6^ cells/kg injected intraperitoneally every 2 weeks (5 injections)	Decreased body weight Reduced hyperlipidemia Improved obesity-associated metabolic syndromes	[36]
Human	HFD mice	4.2 × 10^7^ cells/kg injected intraperitoneally (second injection after 10 weeks)	Reduced blood glucose levels and improved glucose tolerance Reduced the levels of inflammatory mediators	[39]
Human	HFD mice	Metformin preconditioned ADMSCs; 5 × 10^5^ cells injected intramuscularly	Decreased body weight Decreased hyperglycemia and enhanced glucose uptake in muscle	[40]
Human	HFD mice	5 × 10^5^ cells injected intramuscularly	Decreased oxidized LDL and IL6 Decreased insulin resistance	[43]
Human	HFD and db/db mice	2 × 10^6^ cells injected intravenously	Suppressed an increase in body weight Improved dyslipidemia	[37]
Rat	HFD rats	2 × 10^6^ cells transplanted intrahepatic	Improved liver function, reducing lipid accumulation Reduced lipid metabolism and oxidative stress	[61]
Human	T2DM mice (HFD + low-dose STZ)	ADMSCs differentiated into IPCs; 1.5 × 10^6^ cells transplanted underneath the kidney capsule	Lowered blood glucose level by increasing the circulating insulin level and ameliorating metabolic parameters, including IL-6	[47]
Mouse	T2DM mice (HFD + low-dose STZ)	Calcium alginate-encapsulated and transplanted	Restored normoglycemia	[49]
Mouse	T2DM mice (HFD + low-dose STZ)	5 × 10^5^ cells injected intravenously	Increased insulin sensitivity Reduced inflammation Decreased fat content in AT and liver Increased pancreatic β-cell mass	[51]
Rat	T2DM rats (HFD + low-dose STZ)	ADMSCs differentiated into IPCs; 3 × 10^6^ cells injected intravenously	Mitigated hyperglycemia	[48]
Rat	T2DM rats (HFD + low-dose STZ)	3 × 10^6^ cells injected intravenously	Lowered blood glucose level, increased glucose tolerance, and improved insulin sensitivity	[50]
Rat	Long-term T2DM rats (HFD + low-dose STZ)	3 × 10^6^ cells injected intravenously	Demonstrated significant protective effects against long-term T2DM complications by alleviating inflammation and promoting tissue repair	[53]
Mouse	Ath+HFD mice	1 × 10^5^ cells injected into the splenic subcapsule	Restored albumin expression in hepatic parenchymal cells and ameliorated fibrosis in liver	[60]
Mouse	Ath+HFD or HFD mice	1 × 10^5^ cells injected into spleen (second injection after 4 weeks)	Prevented the progression of NASH fibrosis by suppressing IL-17-mediated inflammation	[63]
Human	Mc4r-KO NASH mice + LPS	1 × 10^6^ cells injected intravenously	Decreased serum alanine transaminase levels and inflammatory markers Improved fibrosis and inflammation	[64]
Human	Mice with liver injury (CCl4)	ADMSCs differentiated into HLCs; 1 × 10^6^ cells injected intravenously	Restored liver function	[65]
Rat	T2DM rats with liver fibrosis (HFD, low-dose STZ + CCl4)	2 × 10^3^ cells injected intravenously (second injection after 2 weeks)	Reduced hyperglycemia and insulin resistance Alleviated liver injury by improving liver function	[62]

## 4. Genetic Modifications of ADMSCs

Despite the positive results obtained in some pre-clinical studies, the actual clinical efficacy of ADMSCs still remains controversial [69,70,71,72]. Several investigations have shown that transplanted ADMSCs present a meager rate of survival and proliferation, possibly because of the damaged microenvironment of the affected tissues, which could lead to cell death [73]. Therefore, there is a need for novel approaches to generate more functional ADMSCs with enhanced therapeutic potential. In this context, genetic manipulation has emerged as a promising strategy.

**Figure 2:**
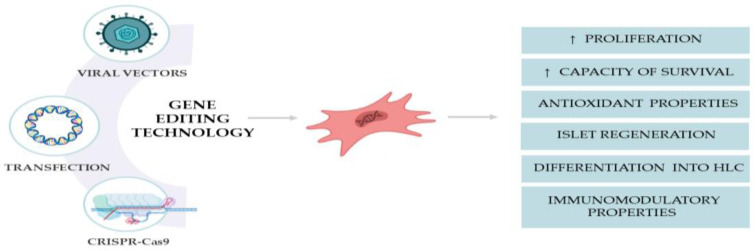
The genetic modification of ADMSCs increases their therapeutic potential by enhancing important characteristics such as proliferation or immunomodulation. Abbreviation: HLCs, hepatocyte-like cells.

The genetic engineering of ADMSCs has been studied in past years to enhance the therapeutic potential of these cells and improve the outcomes after transplantation. Several genetic engineering methods have been described to modify the ADMSC gene expression profile. These techniques can be broadly classified as viral- and non-viral-based transfection as well as Clustered Regularly Interspaced Short Palindromic Repeats (CRISPR)-Cas9 gene editing technology (Figure 2).

### 4.1. Viral-Based Transfection (Transduction)

Viral vectors are the most used gene transfer tool to modify the MSC genome due to their high efficiency in DNA transfer compared to non-viral methods. This gene-editing technique ensures the stable and long-term transcription of the target gene and, consequently, better efficiency than other methods [74]. In addition, the high efficiency of viral transduction does not modify the cell differentiation capacity or the immunophenotypic characteristics of ADMSCs [75].

Currently, lentivirus and adenovirus are the most predominant vectors used for ADMSC transduction. They have been used to enhance different properties of ADMSCs, such as proliferation or differentiation, thus improving their therapeutic capacity. For instance, Cho et al. overexpressed CXCR4 in ADMSCs using lentiviral vectors [76]. CXCR4 is a G-coupled transmembrane glycoprotein that mediates the stromal-derived factor-1 (SDF-1) signaling pathway and plays essential roles in the migration, engraftment, and proliferation of stem cells [77]. They reported that CXCR4 transduction into ADMSCs offered protection against induced cell death while increasing cellular growth and migration. These results suggest that the genetic modification improved ADMSC motility, retention, and proliferation, which could benefit in vivo migration, expansion, and therapeutic efficiency.

Other investigations focused on the enhancement of the antioxidant properties of ADMSCs by overexpressing key antioxidant enzymes. Baldari et al. demonstrated that the overexpression of SOD2 by lentiviral transduction significantly increased the survival rate of ADMSCs when exposed to hypoxic conditions [78]. Similar experiments were performed by Sen et al. but, in this case, using an adenoviral vector to overexpress SOD2. When these modified ADMSCs were exposed to high glucose, a reduction in superoxide generation and inflammation were observed compared with control cells, thus promoting ADMSC survival [79].

Another approach is to use viral vector-modified ADMSCs as a vehicle to deliver proteins that would otherwise be deficient in a specific condition. Sun et al. overexpressed betatrophin (BET), a hormone secreted by the liver and AT that stimulates pancreatic β-cell proliferation, in ADMSCs [80]. They reported that the overexpression of BET did not affect ADMSC proliferation or differentiation. However, the co-culture of human islets with modified ADMSCs did induce islet proliferation, β-cell specific transcription factor expression, and glucose-dependent insulin production. Thus, BET overexpression could be an innovative strategy for inducing β-cell regeneration and an alternative to insulin injections by increasing the number of endogenous insulin-producing cells in patients with diabetes.

The potential of lentiviral vectors to induce the transdifferentiation of ADMSCs has also been explored. Davoodian et al. overexpressed miR-122 and silenced let-7f, two key microRNAs in hepatic differentiation, in ADMSCs [81]. This modification resulted in the increased expression of hepatocyte markers, including ALB, AFP, CK18, CK19, and HNF4a, as well as urea, albumin, and glycogen production, confirming that the modified cells differentiated toward HLC. Therefore, these results demonstrate the possibility of using viral vectors to induce the transdifferentiation of ADMSCs.

The results so far propose viral vectors as an efficient tool to modify ADMSCs and increase their therapeutic potential. However, it has been reported that viral expression systems can cause immune and inflammatory responses in the host, and viral insertion in the host’s genome poses a tumorigenic risk [82]. Accordingly, safety is the most significant barrier to future clinical therapeutic applications of modified ADMSCs.

### 4.2. Non-Viral Methods

To bypass the safety concerns associated with viral vectors, alternative, non-viral-based methods for transgene delivery have been established for ADMSCs. Currently, the non-viral genetic modification of ADMSCs can be performed by physical methods such as electroporation, nucleofection, and sonoporation, or chemical methods including lipidic agents, polymers, and inorganic nanoparticles [83].

Non-viral methods have been efficiently used to enhance the proliferation and differentiation capacity of ADMSCs. Sox2 and Oct4 are transcription factors that maintain pluripotency in embryonic stem cells, enhance proliferation, and prevent cell senescence in ADMSCs. Han et al. overexpressed Sox2 and Oct4 in ADMSCs by liposomal transfection [84] and showed that the modified cells exhibited enhanced proliferation and adipogenic differentiation capacity. Similar results were obtained by Kim et al. after overexpressing the proliferation regulator micro-RNA miR-302 [85]. They also observed an increase in the proliferation and cell survival of modified ADMSCs. In general, non-viral modification has shown benefits in the proliferation capability of ADMSCs, however, more research needs to be performed before clinical translation due to their lower efficiency.

### 4.3. CRISPR/Cas9 System

In recent years, new genetic modification tools have emerged in order to promote the insertion, deletion, or correction of genes at specific sites in the genome. Due to its high efficiency, specificity, simple design, and cost-effectiveness, CRISPR/Cas has become the most widely used genome editing technology [86,87]. The CRISPR/Cas9 system is based on the nucleolytic activity of the endonuclease protein Cas9, which is conducted to a specific site in the genome by a guide RNA (gRNA). The Cas9 protein binds to the target site determined by the gRNA and performs a double-strand break (DSB) followed by the introduction of mutations at the cleavage site by the non-homologous end-joining repair mechanism of the cell. Using a predesigned repair template, this system can not only knock out genes but also knock in (CRISPR/Cas9-SAM-System) or even insert specific mutations [86,88,89].

The efficiency of the CRISPR/Cas9 system has already been tested in MSCs by targeting critical genes involved in adipocyte differentiation and function. Lundh et al. introduced the CRISPR/Cas9-SAM system in MSCs through viral delivery and used gRNA targeting *Pparg2, Prdm16, Zfp423*, or *Ucp1* [90]. They demonstrated that this system could efficiently manipulate gene expression in pre- and mature adipocytes in vitro. This gene editing technology has also been optimized in ADMSCs obtained from human AT. Kamble et al. developed a method for gene knockout by introducing the CRISPR/Cas9 system through electroporation in ADMSCs. They knocked out *PPARG* genes with more than 90% efficiency, blocking the differentiation of ADMSCs into adipocytes [91].

Further exciting research using the CRISPR/Cas9 system on ADMSCs was performed by Claussnitzer et al. who targeted *ARID5B*, a gene essential for adipocyte development and normal lipid metabolism [92]. They identified a single nucleotide mutation in *ARID5B* that results in a reduction in mitochondrial thermogenesis and an increase in lipid storage in adipocytes. Using the CRISPR/Cas9 system, they repaired the *ARID5B* motif in ADMSCs from a patient with the risk allele, restoring thermogenesis and lipid metabolism [93].

Hence, CRISPR/Cas9 gene editing in ADMSCs seems to be a promising tool for therapeutical applications. However, all these investigations are still in an early stage, and more positive results are needed for their translation into the clinic.

## 5. Genetic Modification of ADMSCs as a Therapeutic Strategy

To successfully translate ADMSCs into clinical practice, it is essential to improve the functionality of these cells, enhancing their capacity for survival, homing to the inflammation site, and immunomodulatory proprieties. Gene editing tools are becoming one of the better ways to achieve this goal. To date, multiple results show the higher in vivo efficiency of genetically modified ADMSCs compared to wild-type ADMSCs to treat metabolic disorders. These investigations are summarized in Table 2.

### 5.1. Genetically Modified ADMSCs in the Treatment of Metabolic Complications Associated with Obesity

Different approaches have been explored to enhance the antioxidant properties of ADMSCs to increase their survival. Briefly, Baldari et al. compared the administration efficiency of will-type ADMSCs versus ADMSCs overexpressing SOD2, an antioxidant enzyme [78]. They proved that modified ADMSCs showed higher cell engraftment and improved oxidative stress resistance, enhancing cell therapy potential. To evaluate the therapeutic capacity of this cytoprotective effect conferred by SOD2 overexpression, Sen et al. administrated these cells to an obese (db/db) diabetic mouse model [79]. They confirmed that mice receiving modified ADMSCs exhibited reduced adiposity and improved glucose tolerance, through anti-oxidative and anti-inflammatory mechanisms, compared to those receiving wild-type cells. Similar results were obtained by Domingues et al., who jointly upregulated the expression of SOD2 and catalase in ADMSC [94]. Taken together, these results suggest that overexpression of antioxidant enzymes using ADMSCs as gene delivery vehicles attenuates AT inflammation and improves glucose tolerance in vivo, proposing ADMSC-mediated gene therapy as a novel and safe therapeutic tool to combat the effects of hyperglycemia derived from obesity.

Another attractive target is neuregulin (Nrg4), a growth factor secreted by AT that regulates lipogenesis in the liver and is reduced in obesity. Wang et al. explored the therapeutic potential of this protein by overexpressing Nrg4 in ADMSCs and then transplanting the modified cells in HFD-fed mice [95]. Their results showed that Nrg4 upregulation could enhance the efficiency of ADMSCs in reducing IR and other obesity-related metabolic disorders, mainly by suppressing inflammation, enhancing glucose uptake in AT and muscle, and attenuating hepatic lipogenesis. It would provide a new therapeutic strategy for treating obesity, IR, and T2D.

### 5.2. Genetically Modified ADMSCs in Diabetes

ADMSCs have immense potency in curing T2DM due to their easy isolation, multiple differentiation potential, and immunomodulatory property. However, despite the promising efficacy in pre-clinical animal models, the administration of ADMSCs does not show clinically satisfactory therapeutic results, varying significantly between people with T2DM [96]. After transplantation in people with diabetes, ADMSCs are faced with an inflammatory and hyperglycemic environment that severely reduces cell viability [96,97], leading to the unsatisfactory efficiency of ADMSCs in diabetic models. However, many gene editing strategies have been adopted to maximize ADMSC therapeutic efficiency.

One interesting approach is using ADMSCs as a vehicle to deliver deficient proteins in diabetic individuals. In this sense, Sun et al. engineered ADMSCs to overexpress BET, a hormone that can increase the production and expansion of insulin-secreting β-cells [80]. Those engineered ADMSCs were then administrated into STZ-induced diabetic mice. This innovative approach increased the ratio of β-cells per islet and improved insulin secretion, ameliorating the hyperglycemia associated with this condition. Another strategy to enhance the therapeutic efficiency of ADMSCs in diabetes treatment is via their differentiation into IPC before transplantation. Pancreatic and duodenal homeobox 1 (*Pdx1*) is an exciting target gene as it plays a crucial role in normal pancreas development and is required for maintaining the normal function of islets. With this in mind, Lin et al. proved that the overexpression of *Pdx1* led to the differentiation of ADMSCs to IPC [98]. Subsequent transplantation of these cells under the kidney capsule of STZ-induced diabetic rats resulted in lowered blood glucose and increased glucose tolerance. Kajiyama et al. performed similar studies, where they transplanted *Pdx1* transduced-ADMSC in a hyperglycemic mouse model with pancreatic damage [99]. They reported that modified cells engrafted properly in the pancreas, wherein they expressed insulin, decreased blood glucose levels, and increased survival. Some years later, Lee et al. performed similar experiments and observed reduced blood glucose levels, although modified ADMSC administration did not restore normoglycemia [100].

A further option to treat diabetes is the xenotransplantation of porcine islets, but this is primarily limited because of immune rejection and the early loss of transplanted islet cells [101]. To overcome this problem, Lee et al. proposed to co-transplant islets with genetically modified ADMSCs overexpressing both heme oxygenase- 1 (HO-1) and the soluble fusion protein of TNF-α receptor (TNF-αR-Fc) [102,103]. HO-1 is a stress-activated inducible enzyme and TNF-αR-Fc suppresses TNF-α induced inflammatory reactions. Both have been described to reduce the deleterious effects of oxidative stress, apoptosis, and the inflammatory factor in many cell lines [104,105]. This double overexpression strategy confirmed that the modified ADMSCs reduced the inflammatory reaction and improved the viability of the transplanted islets, significantly reducing rejection and reversing hyperglycemia.

### 5.3. Genetically Modified ADMSCs in NAFLD

Multiple studies have demonstrated that modified-ADMSC administration in obesity mice models has systemic benefits, reducing lipid deposition in the liver which is the leading cause of the development of NAFLD. For instance, Nrg4 overexpressing-ADMSCs reduced the liver fat content by attenuating hepatocyte lipogenesis [95]. In addition, antioxidant-upregulated ADMSC delivery has been shown to reduce fat accumulation in the liver by reducing systemic inflammation [94]. Domingues et al. demonstrated that mice receiving SOD2 and catalase-overexpressing ADMSCs showed lower levels of steatosis than those transplanted with non-modified ADMSC.

Nevertheless, the therapeutic potential of modifying ADMSCs has been tested not only for early stages of NAFLD derived by obesity-like steatosis but also for more advanced phases such as fibrosis. For instance, Kang et al. generated FGF21-secreting ADMSC and administrated them to a liver fibrosis mice model. The transplantation of FGF21-ADMSC significantly improved liver fibrosis by decreasing serum hyaluronic acid and reducing fibrosis-related factors’ expression [106]. Similarly, Lou et al. established ADMSC overexpressing miR-122, a key regulator of liver fibrosis. They observed that administering these cells to mice improved liver fibrosis by suppressing the activation of HSC and alleviating collagen deposition [107].

### 5.4. Genetically Modified ADMSCs in Metabolic Syndrome

In the last few years, activating brown adipocytes to increase energy expenditure has emerged as a promising treatment strategy for MetS. Several studies have proved that the implantation of brown fat into obese mice improves glucose tolerance. However, the translation to humans has been limited by the low abundance of primary human beige adipocytes [108,109]. To overcome this limitation, research has focused on the potential of ADMSCs obtained from scWAT, which is much more abundant, and its genetic modification to turn into brown-like adipocytes (“browning”).

In this context, Wang et al. generated human brown-like cells from ADMSCs using CRISPR/Cas9-SAM-gRNA to activate the endogenous expression of uncoupling protein 1 (UCP1), the main thermogenesis regulator. Obese mice transplanted with these modified cells showed a significant improvement in glucose tolerance, insulin sensitivity, and increased energy expenditure [110]. With the same goal, Tsagkaraki et al. tried to target the nuclear receptor-interacting protein 1 (NRIP1), a transcriptional co-repressor that regulates energy metabolism and suppresses thermogenesis [111]. They also used CRISPR technology to disrupt NRIP1 in ADMSCs, notably increasing thermogenesis in these cells. The transplantation of these CRISPR-enhanced brown-like adipocytes into HFD-fed mice decreased adiposity and liver fat deposition while enhancing glucose tolerance compared to the implantation of unmodified adipocytes.

These results demonstrate the benefits of using CRISPR/Cas9 technology to engineer human white adipocytes to display phenotypes similar to brown fat and may open cell-based therapeutic opportunities to combat metabolic disorders caused by high-calorie diets. Furthermore, CRISPR-based therapy is a safe alternative, as ex vivo delivered Cas9 and sgRNA are entirely degraded by human cells after high-efficiency genomic modification without detectable off-target editing.

**Table 2:** Relevant genetic modifications of ADMSCs to increase their therapeutic potential.

Modification in ADMSC	Method of Modification	ADMSC Source	Model	Effects	References
SOD2 overexpression	Lentivirus	Human	7 × 10^5^ cells injected subcutaneously into mice	Promoted the survival and engraftment of transplanted ADMSCs	[78]
SOD2 overexpression	Adenovirus	Human	Intraperitoneal injection in db/db mice	Reduced body weight Improved glucose tolerance Reduced inflammation	[79]
SOD2 and catalase overexpression	Adenovirus	Human	1.5 × 10^6^ cells injected intraperitoneally in HFD mice	Reduction in liver fat content Reduced systemic inflammation	[94]
NRG4 overexpression	Lentivirus	Mouse	2 × 10^6^ cells injected intravenously into HFD mice	Reduced blood glucose levels and enhanced insulin sensitivity Decreased fat cell deposition and lowered TG and TC levels in the serum and liver	[95]
UCP1 overexpression	CRISPR–Cas9	Human	Subcutaneous implantation into HFD mice	Improved glucose tolerance and insulin sensitivity and increased energy expenditure	[110]
NRIP1 deletion	CRISPR–Cas9	Human and mice	Subcutaneous implantation into HFD mice	Decreased adiposity and levels of liver triglycerides Enhanced glucose tolerance Decreased liver triglycerides	[111,112]
BET overexpression	Adenovirus	Human	1 × 10^6^ cells injected intravenously into T2DM mice	Ameliorated hyperglycemia and weight loss Induced β-cell proliferation and insulin secretion	[80]
PDX1 overexpression	Retrovirus	Mouse	5 × 10^5^ cells injected intravenously into T2DM mice	Decreased blood glucose levels and increased survival	[99]
PDX1 overexpression	Adenovirus	Human	2 × 10^6^ cells transplanted under the renal capsule of T2DM mice	Reduced blood glucose levels, although they did not restore normoglycemia	[100]
PDX1 overexpression	Lentivirus	Human and rat	2 × 10^6^ cells transplanted under the renal capsule of T2DM rats	Reduced blood glucose levels and led to higher glucose tolerance, smoother fur, and fewer cataracts	[98]
HO-1 and TNF-αR-Fc overexpression	Adenoviral	Human	Transplantation of porcine islets with ADMSC in T1DM mice	Reversed hyperglycemia	[103]
FGF21 overexpression	Plasmid transfection	Not defined	1.5 × 10^6^ cells injected intravenously into TAA-treated mice	Improved liver fibrosis	[106]
MiR-122 overexpression	Lentiviral	Mouse	1 × 10^5^ cells injected intravenously into CCL4-treated mice	Decreased liver fibrosis	[107]

## 6. Clinical Trials with Modified ADMSCs

The promising pre-clinical results have encouraged researchers to test MSC therapy in human clinical trials. In the last decade, more than 400 clinical trials based on MSCs have been performed to treat various diseases, according to the US National Institute of Health–Clinical Trials database (http://clinicaltrials.gov, accessed on 1 March 2020). Regarding the metabolic complications of obesity, interesting results have been obtained with MSCs obtained from different sources to treat T2DM. Zhang et al. carried out a systemic review and meta-analysis of published clinical trials to evaluate the safety and efficacy of MSC therapy for T2DM [113]. They considered 11 T2DM studies including 386 patients, all treated with MSCs isolated from BM, placenta, or UC. This analysis showed that stem cell therapy improved insulin requirements and had favorable therapeutic effects, but it is unclear which source of MSCs is most suitable for treating the disease. However, clinical studies using ADMSCs to treat T2DM are scarcer [96,113]. One ongoing study using ADMSCs found that fasting plasma glucose and glycated hemoglobin (HbA1c) in ADMSC-treated diabetic individuals decreased more than the controls (NCT00703612 ClinicalTrials.gov).

MSC therapy has also been used for liver disease therapy. Currently, MSCs applied for liver disease in the clinic are primarily obtained from UC and BM, and only in a few cases from AT [23]. For instance, Sakai et al. conducted a clinical trial using autologous ADMSCs to treat patients with liver cirrhosis derived from NASH or fatty liver [114,115]. They observed a significant improvement in serum albumin concentration and pro-thrombin activity in most of the treated patients. However, only seven patients were in the trial, so more extensive trials are warranted to confirm the therapeutic efficiency.

Most of the clinical trials based on MSC therapy conducted to date have used autologous MSCs to minimize the immune response [116,117]. However, ADMSCs isolated from patients that have a chronic inflammatory disease such as obesity have less therapeutic potential compared with metabolically healthy individuals [118]. Furthermore, after being transplanted into obese individuals, MSCs are faced with an inflammatory environment that impairs their survival, leading to non-ideal therapeutic effects [119]. This fact supports the need to increase the therapeutic potential of ADMSCs, and one of the more promising strategies to achieve that is genetic modification.

To date, genetically engineered MSCs are being tested in only a few clinical studies to treat different conditions [75,120,121]. Promising results have been obtained with modified MSCs to treat some oncological diseases. Thus, genetically modified MSCs have been used in clinical trials of advanced gastrointestinal cancer [122], ovarian cancer (NCT02530047 ClinicalTrials.gov), lung cancer (NCT03298763 ClinicalTrials.gov), or neck cancer (NCT02079324 ClinicalTrials.gov). Regarding metabolic diseases, to the best of our knowledge, there is only one phase 1 clinical trial started in 2022 based on CRISPR-modified-MSC for T1DM (NCT05210530 ClinicalTrials.gov).

## 7. Limitations and Side Effects of ADMSC Therapy

Although pre-clinical trials of ADMSC therapy are effective and have few side effects, many problems still need to be solved before ADMSCs can be efficiently applied in the clinic for metabolic disorders. First, a standardized protocol needs to be developed, including specifications such as the injection site, injection method, injection dose, and other variables. The different conditions used so far could explain the discrepancies between studies and makes it difficult to evaluate the efficiency of the treatment.

Second, it is critical to address whether autologous or allogeneic ADMSC therapy is safer and more efficient for metabolic complications. While autologous ADMSCs may be the better option to avoid the immune response, factors including donor comorbidities such as obesity or diabetes may compromise the therapeutic potential of these cells [123]. Conversely, allogeneic ADMSC therapy may be more efficient but may cause an immune response [124]. It has been described that allogeneic ADMSCs are recognized by both the innate and adaptative immune systems and that their viability may be decreased following immune recognition. Moreover, some studies have shown that an antibody response is generated after the treatment, which may inhibit ADMSCs’ therapeutic efficacy upon repeat injections [125,126].

Regarding the immune response, it is also essential to consider the possible changes in the immunogenicity of ADMSCs during their differentiation to HLC or IPC [127]. For instance, Li et al. described the upregulation of HLA-DR on MSCs after hepatocyte differentiation, which induced significantly more CD3+ and CD45+ cells after transplantation compared to undifferentiated MSCs [128]. Similarly, Mohammadi et al. reported that IPC exhibited an increased expression of MHC-I and CD80 that induced the proliferation of splenocytes, activation of CD4+ T cells, and IFNγ response [129,130,131]. Therefore, strategies such as genetic modification to reduce this immunogenicity would be extremely important for the future development of such therapies.

In addition to the limitations already discussed, the genetic stability of ADMSCs after expansion and manipulation is another major issue. Multiple replications in vitro expose cells to the risk of accumulating genetic and epigenetic alterations, which may promote cell senescence or even cell transformation, thus possibly affecting treatment efficacy and patient safety [132]. Moreover, genetic modification may increase this risk, as gene therapy vectors can integrate into the host’s genome outside the target sequences, promoting tumorigenesis [74]. Thus, there is a need to optimize this technology prior to its use in clinical routine, especially considering its effectiveness, safety, and specificity.

## 8. Conclusions

Considering the good results obtained in vitro and the positive outcomes in clinical trials with modified cells, it is rational to think that modified ADMSCs are a promising strategy for treating obesity and its comorbidities. However, as obesity is a multifactorial disorder that affects metabolic homeostasis globally, it is critical to identify the specific cells/tissues to target using gene therapy-based ADMS. Consequently, additional pre-clinical studies are needed to ensure the safety and demonstrate the therapeutic potential of engineered ADMSCs.

## Data Availability

Not applicable.
